# Evaluation of the relationship between ovarian reserve with congenital anomalies and intramural uterine leiomyoma among infertile women: a cross-sectional study

**DOI:** 10.1186/s13048-023-01149-7

**Published:** 2023-04-06

**Authors:** Ashraf Moini, Mehri Kalhor, Shahideh Jahanian Sadatmahalleh, Maryam Niknejadi, Malihe Nasiri, Azar Yahyaei, Shohreh Irani, Seyedeh Saeedeh Mousavi, Saeideh Mikaeili, Negin Mirzaei

**Affiliations:** 1 Breast Disease Research Center (BDRC), Tehran University of Medical Sciences, Tehran, Iran; 2 Department of Obstetrics and Gynecology, Arash Women’s Hospital, Tehran University of Medical Sciences, Tehran, Iran; 3 Department of Endocrinology and Female Infertility, Reproductive Biomedicine Research Center, Royan Institute for Reproductive Biomedicine, ACECR, Tehran, Iran; 4 Kowsar hospital, Qazviin University Medical of Science, Qazvin, Iran; 5 Department of Reproductive Health and Midwifery, Faculty of Medical Sciences, Tarbiat Modares University, Tehran, Iran; 6 Department of Reproductive Imaging, Reproductive Biomedicine Research Center, Royan Institute for Reproductive Biomedicine, ACECR, Resalat highway, end of North Bani Hashem St., East Hafez St., Royan institute, Tehran, 1665659911 Iran; 7 Department of Basic Sciences, Faculty of Nursing and Midwifery, Shahid Beheshti University of Medical Sciences, Tehran, Iran; 8 Robinson Research Institute, Faculty of Health and Medical Sciences, University of Adelaide, Adelaide, SA Australia

**Keywords:** Ovarian reserve, Uterine Leiomyoma Uterus congenital anomaly, Infertility

## Abstract

**Background:**

Ovarian reserve is a crucial indicator of a woman’s fertility potential, which is determined by the quality and quantity of antral follicles and oocytes. However, certain factors such as endometriosis, pelvic inflammatory disease, myoma, and the natural process of aging can lead to a poor ovarian response to stimulation, reducing a woman’s chances of conceiving.

**Objective:**

To evaluate the effect of uterus congenital anomalies and uterine leiomyoma are associated on ovarian reserve.

**Methods:**

The present cross-sectional study was performed on 321 infertile women in three groups consisted of 97 infertile women with intramural uterine leiomyoma and 81 infertile women with uterine anomalies and 143 infertile women without uterine anomalies and uterine leiomyoma during 2017–2019 in Royan Center. Sampling method was continuous and available. Data collection tool in this study was a questionnaire which was in two parts of individual variables and the second part was related to ultrasound results (number of antral follicles and ovarian volume) and laboratory tests (Anti-Mullerian Hormone (AMH) and Follicle-stimulating Hormone (FSH)). Ovarian reserve parameters were measured in three groups on the third day of the cycle in both groups. Data analysis was performed using SPSS software version 21. Quantitative variables were analyzed using t-test, qualitative variables were analyzed using chi-square test.

**Results:**

The results of in laboratory parameters showed that there was no statistically significant difference between the three groups in FSH (2.35 ± 1.55, 2.07 ± 1.81, 2.31 ± 1.93) and AMH (6.84 ± 2.75,7.52 ± 3.14,6.93 ± 3.04), respectively (P > 0.05). The results of sonographic variables also showed that the variables include number of antral follicles in right ovarian, number of antral follicles in left ovarian have statistically significant between the three groups (5.73 ± 2.69,4.84 ± 3.14,6.66 ± 3.13), respectively (P < 0.05).

**Conclusion:**

The results of the present study showed that uterine abnormalities and uterine leiomyoma with different mechanisms such as reduce of antral follicle numbers and the effect on uterine and ovarian blood flow lead to a decrease in ovarian reserve and infertility. Therefore, treatment and surgery can reduce these effects and improve the fertility of the affected women.

**Supplementary Information:**

The online version contains supplementary material available at 10.1186/s13048-023-01149-7.

## Introduction

Infertility is a major issue affecting around 21% of couples worldwide and is a common concern among patients [1]. Over the past 15 years, studies on fertility decline and its clinical consequences have led to efforts of the ovarian reserve measure in infertile patients [2]. Ovarian reserve is a measure of a woman’s fertility potential that is related to the number of antral follicles and oocyte quality [3]. Ovarian reserve is usually measured by describing the size and quality of the remaining ovarian follicular. Various methods have been proposed to predict future fertility and the likelihood of successful infertility treatment by assessing the size and quality of remaining ovarian follicles. Given the importance of oocyte numbers for successful IVF cycles, finding effective fertility treatments is critical [4].

Reduced ovarian reserve can lead to a diminished follicular response to gonadotropin stimulation and fewer eggs being produced [5]. Poor ovarian response to stimulation may be due to aging, endometriosis, myoma, and pelvic inflammatory disease. Uterine leiomyoma is diagnosed in 1.5% of infertile women. In addition, in 2.4% of women with infertility, fibroids are the only diagnostic findings. The association of subserosal and intramural Uterine Leiomyoma with infertility is discussed [6]. The mechanism of the effect of Uterine Leiomyoma on fertility is not known, but theories includes mechanical blockage of the fallopian tubes, abnormal fetal and sperm transfer, abnormal arteries, abnormal endometrial development, chronic inflammation of the uterine cavity, non-abnormal endocrine environment androgen is in the endometrium [7]. Additionally, the adverse effects of uterine leiomyoma on IVF/ICSI outcomes and its role in fertility have been reported [8].

Uterine abnormalities, both congenital and acquired, can significantly impact reproductive outcomes. Congenital uterine abnormalities, in particular, have been linked to both positive and negative reproductive outcomes [9]. For example, the prevalence of uterine malformations in infertile women is 3.5%, which is 21 times higher than in women with normal fertility.

On the other hand, congenital uterine abnormalities affect uterine function and have been associated with normal and adverse reproductive outcomes. The prevalence of uterine malformations in infertile women is 3.5%, which is 21 times higher than in women of normal fertility [9]. The uterus has a role in a mechanical support of the ovary, and its removal may adversely affect ovarian function and consequently reduce ovarian reserve [10]. In addition, congenital anomalies may also interfere with normal implantation and placenta formation and they cause infertility and premature miscarriage [11]. Uterine abnormalities can cause miscarriage, preterm labor, and difficult labor [12].

Considering the effect of congenital anomalies and uterine leiomyoma in infertility, evaluation of these factors on ovarian function which is the main key in counseling infertility patients should be considered. Also we should be aware of ovarian reserve to predict ovarian response in different methods of ovulation stimulation, greatly helped to predict the success of each ovulation stimulation cycle, and prevents the cancellation or failure of stimulation cycle, spending additional costs, waste of time and most importantly the mental and psychological problems of patients.

The aim of this study is to evaluate the impact of congenital uterine abnormalities and uterine leiomyoma on ovarian reserve. In particular, the study will focus on the effects of intramural uterine leiomyoma, as previous research has shown that submucosal leiomyoma can impact endometrial receptivity, whereas subserosal leiomyoma does not [6, 8], therefore, when referring to uterine leiomyoma in this study, we specifically mean intramural uterine leiomyoma.

## Materials and methods

This cross-sectional study was conducted on women categorized into three groups: group 1 consisted of infertile women with uterine anomalies, group 2 included infertile women with uterine leiomyoma, and group 3 comprised infertile women without uterine leiomyoma and uterine anomalies. The study was conducted at Royan Center between 2017 and 2019, and the Research Ethics Committee of Royan Institute approved the study protocol (code: IR.ACECR.ROYAN.REC.IR.ACECR.ROYAN.REC.1395.6). The inclusion criteria for the study were women under the age of 37, with intramural uterine leiomyoma or uterine abnormalities (septal uterus, bicorn uterus, double uterus, walled uterus, Rocky-Tansky syndrome), no history of surgery on the uterus and ovaries, no recurrent miscarriage, and not obese (body mass index less than 30). Exclusion criteria were endometriosis and a history of thyroid disease, diabetes, or autoimmune disorders.

The sampling method was continuous and available, with eligible participants added sequentially to the case group throughout the study. Prior to participation, written consent forms were obtained from all patients, and the study procedures were explained to them. Participants were informed about the research goals, methods, potential benefits and risks, as well as any potential inconvenience they may experience. They were given the right to choose whether or not to participate in the research and were assured that they could withdraw at any time.

The appropriate formula was used to calculate the sample size, and it was determined that a sample of 310 women was required, with a 10% sample loss, at a 95% confidence interval and an error of 3 (d).

Sample size estimation formula:

$$n = n \ge 2\frac{{{{\left( {{z_{\alpha /2}} + {z_\beta }} \right)}^2}{\sigma ^2}}}{{{{\left( {{\mu _1} - {\mu _2}} \right)}^2}}} = 310$$

**Fig. 1:**
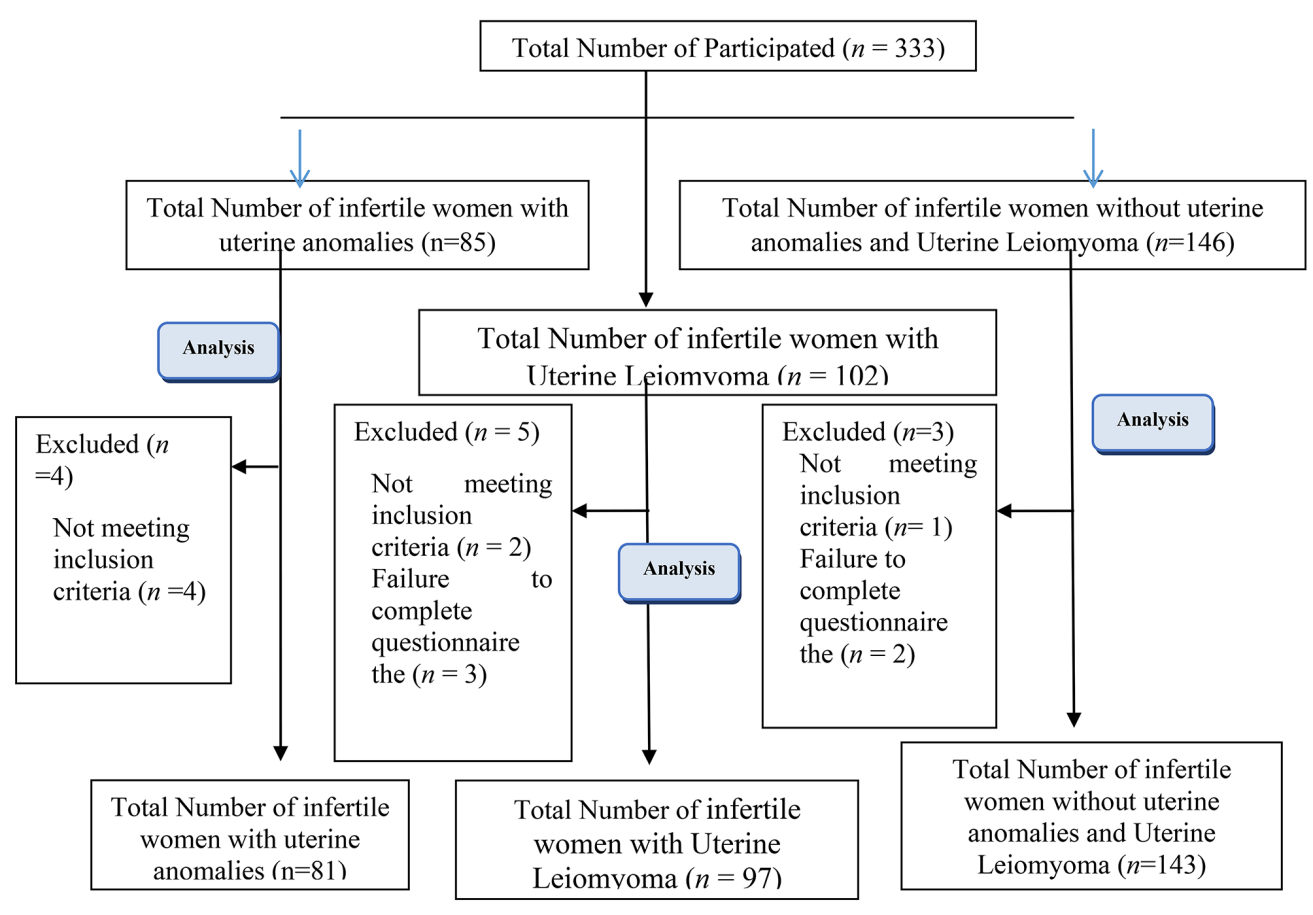
Flow chart for this cross-sectional study

Initially, 333 women were enrolled in the study, with 85 women in the infertile group having congenital uterine abnormalities, 102 women having uterine leiomyoma, and 146 women without uterine abnormalities or leiomyoma. Twelve women were subsequently excluded due to failure to meet the inclusion criteria, resulting in a total of 321 women being included in the study (Fig. 1). The participants were divided into three groups: 97 infertile women with intramural uterine leiomyoma, 81 infertile women with uterine anomalies, and 143 infertile women who were healthy and only their spouse had infertility problems without uterine abnormalities or leiomyoma.

The data collection tool consisted of a self-made research questionnaire that was divided into two parts. The first part dealt with individual variables, while the second part focused on laboratory and ultrasound tests. On the third day of the menstrual cycle, levels of FSH and AMH were measured to assess ovarian reserve in all three groups. FSH blood levels were determined using the radioimmunoassay method in the Royan laboratory. Serum AMH levels were measured using the ELISA method and a kit (AMH-EIA; Beckman Coulter, Marseilles, France) in accordance with the manufacturer’s instructions. The number of antral follicles and ovarian volume were determined using ultrasound, after which ovarian reserve was compared between the three groups.

Data analysis was performed using SPSS software version 21. The variables were analyzed using t-test, chi-square test, Mann-Whitney test, and covariance test. Confounding variables such as age and type of infertility were adjusted using logistic regression analysis. Quantitative variables were analyzed using t-test, while qualitative variables were analyzed using chi-square test and Mann-Whitney test. Adjustments for confounding variables were also made using logistic regression and covariance tests.

## Results

The study included 321 infertile women, among whom 97 had intramural uterine leiomyoma, 81 had uterine anomalies including Rocky-Tanks syndrome (1 person), didelphys uterus (13 patients), bicorn uterus (5 patients), unicorn uterus (28 patients), and septum uterus (34 patients), and 143 were infertile but did not have uterine anomalies or uterine leiomyoma.

The characteristics of each group are presented in Table 1. The participants had a mean age of 30.94 ± 4.41 years and a mean Body Mass Index (BMI) of 25.62 ± 3.68. No significant differences were found between the groups in terms of BMI, menstrual bleeding duration, duration of infertility, number of deliveries, number of injected oocytes (I.O), number of in vitro fertilization (IVF) failures, number of intrauterine insemination (IUI) failures, and menstrual status (P > 0.05).

**Table 1:** Demographic characteristics in three groups

Variable	Case Group (Uterine Anomalies) N = 81	Case Group (Uterine Leiomyoma) N = 97	Control Group N = 143	P-Value	
Age^*^	30.94 ± 4.63	31.87 ± 3.44	30.78 ± 4.61	0.18	
BMI (kg/ m²) *	25.63 ± 3.59	25.61 ± 3.08	25.14 ± 3.66	0.51	
During menstrual (day) *	30.03 ± 2.24	28.67 ± 2.80	29.60 ± 2.01	0.09	
Duration of infertility(year)*	3.82 ± 2.41	4.63 ± 2.97	3.86 ± 2.58	0.13	
During bleeding menstrual cycle (day)*	5.63 ± 1.38	5.53 ± 1.33	5.79 ± 1.34	0.489	
Number of delivery**				0.178	
0	85(87.6%)	74(91.4%)	135(94.4%)		
1	11(11.3%)	6(7.4%)	7(4.9%)		
≥2	1(1.0%)	1(1.2%)	1(0.7%)		
Number of IVF fail in previous cycle **				0.47	
0	85(87.6%)	66(81.5%)	121(84.6%)		
1	10(10.3%)	10(12.3%)	17(11.9%)		
≥2	2(2/1%)	5(6/2%)	5(3/5%)		
Number of IUI fail in previous cycle **				0.41	
0	88(90.7%)	77(95.1%)	129(90.2%)		
1	5(5.2%)	3(3.7%)	10(7.0%)		
≥2	4(4.2%)	1(1.2%)	4(2.8%)		
Menstrual status***				0.45	
Regular	85 (87.6%)	72 (88.9%)	132 (92.3%)		
Irregular	12 (12.4%)	9 (11.1%)	11 (7.7%)		
Type of infertility***				0.03	
Primary	77 (79.4%)	75 (92.6%)	126 (88.1%)		
Secondary	20 (20.6%)	6 (7.4%)	17 (11.9%)		

*Values are given as mean ± SD by using t-test test

** Values are given as mean ± SD by using Man-Witni test

*** Values are given as a number (%) by using Chi-square test

BMI: Body mass index

Table 2 shows the results of ANOVA test for comparing the laboratory and ultrasound parameters in the three groups. The results showed no significant difference between the groups for (FSH and AMH tests) (P > 0.05). In ultrasound parameters such as the number of antral follicles in the right ovarian, the number of antral follicles in left ovarian and size of uterine were a significant difference among the groups (P < 0.05). But in endometrial Thickness was not a significant difference (P > 0.05).

**Table 2:** Comparison of laboratory and ultrasound parameters in three groups

variable	Case group (Uterine Anomalies) n = 81	Case group (Uterine Leiomyoma) n = 97	Control group n = 143	P-value*
AMH (ng/mL)	2.35 ± 1.55	2.07 ± 1.81	2.31 ± 1.93	0.35
FSH (mIU/mL)	6.84 ± 2.75	7.52 ± 3.14	6.93 ± 3.04	0.23
Number of antral follicles in right ovarian	5.73 ± 2.69	4.84 ± 3.14	6.66 ± 3.13	**< 0.001**
Number of antral follicles in left ovarian	5.74 ± 2.79	4.68 ± 2.89	6.46 ± 3.12	**< 0.001**
Endometrial thickness	4.46 ± 0.97	4.52 ± 1.14	4.67 ± 1.24	0.38
Uterine size	1.11 ± 0.38	1.84 ± 0.99	1.01 ± 0.17	**< 0.001**

* Covariance test

AMH: Anti-Mullerian Hormone, FSH: Follicle-stimulating Hormone

Table 3 shows the ultrasound parameters’ results after adjusting. In the ultrasound parameters such as the number of antral follicles in the right ovarian and the number of antral follicles in the left ovarian, the results showed a near significant difference among the three groups (P = 0.051). So that, the number of follicles in the right ovary in groups 1 and 2 was less significant than in controls 0.84 and 1.17. The number of follicles in the left ovary in group 2 was lower and more significant than in control 1.10.

**Table 3:** Comparison of ultrasound variables in the case groups to the control group

Dependent VariableParameter		B	Std. Error	t			P-value	95%confidence interval		
								Lower bound	Upper bound	
Number of antral follicles in right Ovarian	Intercept	6.584	0.260	25.36			< 0.001	6.073	7.095	
	[Group1 = Uterine Anomalies]	-0.790	0.403	-1.96			0.051	-1.583	0.003	
	[Group2 = Myoma]	-1.707	0.426	-4.010			< 0.001	-2.545	-0.870	
	[Group3 = Control]	0^a^	.	.			.			
Number of antral follicles in left Ovarian	Intercept	6.416	0.256	25.06			< 0.001	5.912	6.920	
	[Group1 = Uterine Anomalies]	-0.622	0.398	-1.56			0.119	-1.404	0.160	
	[Group2 = Myoma]	-1.811	0.42	-4.31			0.001	-2.637	-0.985	
	[Group3 = Control]	0^a^	.	.				.		
The size of uterine	Intercept	1.015	0.048	21.33			< 0.001	0.921	1.108	
	[Group1 = Uterine Anomalies]	0.099	0.074	1.338			0.182	-0.047	0.244	
	[Group2 = Myoma]	0.825	0.078	10.57			< 0.001	0.671	0.978	
	[Group3 = Control]	0^a^	.	.		.				

*Adjusted by type of infertility

a: This parameter is set to zero because it is redundant

## Discussion

The results of this study demonstrate a significant distinction between infertility and uterine problems, including uterine anomalies and uterine leiomyoma (P < 0.05). Previous research by Akhtar et al. [11] also reveals a correlation between uterine abnormalities, particularly septal uterus, and infertility. Hysteroscopy metroplasty in women with uterine septum can significantly improve fertility [11]. Similarly, Chan et al. [13] found that uterine abnormalities, such as unicorn, uterine bifurcation, Müllerian malformation, uterine agenesis, uterine hypoplasia, and T-shaped uterus, are more prevalent in infertile women (13.3%) than the general population (5.5%) and can affect AMH level and ovarian reserve parameters. In contrast, Hatasaka et al. suggest that uterine anomalies are not associated with infertility but with decreased live birth rates [14].

Infertility in uterine problems may occur through various mechanisms, including cervical incompetence, abnormal uterine contractions, reduced blood flow to the ovaries, and decreased uterine and ovarian volume, which can negatively affect ovarian reserve factors and AMH levels. Therefore, the diagnosis and treatment of uterine abnormalities are critical in reducing infertility [15].

Although no significant difference was found between ovarian reserve markers in this study, previous studies have reported such differences. This could be attributed to the small sample size in this study, and further studies with a larger sample size are recommended. Hur et al. [15] also found that women with infertility and unicorn uterus are more prone to infertility (11%).

Therefore, the treatment of uterine anomalies can reduce infertility and improve fertility status in women with such anomalies [16]. Additionally, congenital uterine anomalies can interfere with normal implantation and placenta formation, leading to infertility and premature miscarriage. Uterine abnormalities with reduced ovarian blood flow can cause a deformity of the uterus, which in turn reduces ovarian reserve [17]. A study found that serum AMH levels were correlated with ovarian volume due to ovarian damage [18]. Therefore, it can be concluded that uterine abnormalities reduce ovarian capacity by affecting ovarian volume or reducing ovarian blood flow.

Pregnancies result from a complex interplay of molecular pathways at the level of female and male gametes during development and their interaction for fertilization, and subsequent embryo development before, during, and after implantation [19]. Inflammatory processes such as peritoneal stretching play a role in cytokine secretion, and the effects of uterine leiomyoma and uterine abnormality can cause stretching of the peritoneum [20].

The present study found a near significant difference between the groups in terms of the number of antral follicles in the right and left ovaries. The number of ovarian follicles in women with uterine problems was lower than in the control group (P = 0.051) [20]. The study by Barbakadze et al. [20] showed that antral follicle count (AFC) is lower in infertile women with uterine anomalies, including uterine leiomyoma and congenital uterine anomalies.

Styer et al. [21] found that women with fibroids had a greater uterine volume, lower serum AMH levels, and fewer antral follicles than women without fibroids. Clinical pregnancy rates were significantly lower in participants with fibroids than in those without uterine fibroids [21]. However, the study by Jayaprakasan et al. [22] showed that uterine anomalies are not associated with a reduction in pregnancy rates following assisted reproductive technology (ART). Finally, the study by Tropeano et al. found no significant changes in mean day 3 FSH and E2 levels, ovarian volume measurements, and antral follicle numbers in women with fibroids [6, 7].

Uterine leiomyoma is a hormonal-dependent pathological condition, and its growth depends on ovarian hormones. Both estrogen and progesterone promote the development of uterine leiomyoma. Factors that increase exposure to estrogen, such as obesity and early menarche, increase the incidence of uterine leiomyoma [23]. Uterine leiomyoma causes an increase in the level of sex hormone-binding globulin (SHBG), leading to an increase in the level of estrogens. This decrease in the number of antral follicles can lead to anovulation infertility. These changes also lead to alterations in hormone receptors that can affect inflammatory responses, and respond to ischemic damage during the menstrual cycle, leading to the initiation of genetic changes (epi) and infertility [24].

The present study shows that there is a decrease in the number of antral follicles in women with Uterine Leiomyoma. The studies suggest that uterine Leiomyoma can be effect on infertility through blood circulation and number of antral follicles. In this way, they affect the blood supply to the ovaries and their fertility [7, 25]. The congenital uterine anomalies effect on fertility with several mechanisms such as: variations in the vascular contribution from the uterine artery and utero-ovarian artery of the contralateral side, extent of the reduction or change of muscular mass of a uterus, degree of cervical competence, and presence and extent of coexistent pelvic disease such as endometriosis [11]. Therefore, according to the evidence and the results of the present study, uterine problems can affect the number of antral follicles in this way.

Studies suggest that uterine leiomyoma affects infertility through blood circulation and the number of antral follicles. This condition affects the blood supply to the ovaries, leading to changes in the size of the uterus and the middle of the luteal phase in women with infertile uterine leiomyoma [26, 27]. Embryo implantation rate and fertility outcomes in women with uterine leiomyoma are affected by uterine deformity mechanism and uterine size change [27]. Therefore, surgical treatments should be performed before fertilization and the use of IVF methods in women with uterine fibroids associated with uterine abnormalities. Laparoscopic myomectomy can improve fertility in women with uterine leiomyoma [28].

Uterine leiomyoma cause deformities of the endometrial cavity, changing the direction of blood flow to the uterine fibroids, which may reduce blood flow to the endometrium, and thus have adverse consequences [29]. Due to the relationship between uterine and ovarian arteries, uterine fibroids with reduced blood flow to the uterus and ovaries can reduce ovarian reserve and cause infertility in women with uterine leiomyoma.

The present study investigated both laboratory and ultrasound ovarian markers and studied women with uterine leiomyoma and uterine abnormalities. The study’s strength is that it increases the quality of fertility and life of women with uterine abnormalities and uterine leiomyoma. However, one limitation of the present study was the small sample size and the need for longer sampling time. Therefore, the researchers recommend carrying out a study with a larger sample size.

## Conclusion

The findings of the present study indicate that uterine abnormalities and uterine leiomyoma can affect fertility through various mechanisms, such as reducing antral follicle numbers and impacting blood flow to the uterus and ovaries, leading to a decrease in ovarian reserve and ultimately resulting in infertility. Therefore, treating and addressing uterine issues can alleviate these effects and improve fertility. It is important for physicians and patients to recognize that ovarian reserve tests should be evaluated in cases of uterine anomalies and uterine leiomyoma. Clinically, identifying the relationship between ovarian reserve markers and these conditions can help to monitor and improve fertility outcomes. While routine ovarian marker testing is not recommended for women with uterine leiomyoma and abnormalities, this study suggests that these women should consider checking their ovarian markers before attempting to conceive.

## Electronic supplementary material

Below is the link to the electronic supplementary material.

Supplementary Material 1

## Data Availability

The data sets used and analyzed for the current study are available upon reasonable request of the corresponding authors Dr. Shahideh Jahanian (shahideh.jahanian@modares.ac.ir) and Dr. Maryam Niknejadi (m.niknejadi@royaninstitute.org).

## List of abbreviations

AMH: Anti-Mullerian Hormone
FSH: Follicle-Stimulating Hormone
IVF: In Vitro Fertilization
ICSI: Intracytoplasmic Sperm Injection
ELISA: Enzyme-Linked Immunosorbent Assay
BMI: Body Mass Index
IUI: Intrauterine Insemination
AFC: Antral Follicle Count
SHBG: Sex Hormone-Binding Globulin
