# Supplementary material for: Evaluation of the relationship between ovarian reserve with congenital anomalies and intramural uterine leiomyoma among infertile women: a cross-sectional study
Source: J Ovarian Res. 2023 Apr 6;16:68. doi: 10.1186/s13048-023-01149-7 (PMC10080793; doi:10.1186/s13048-023-01149-7)
Supplement: Supplementary file 1 — Supplementary Material 1 [file 13048_2023_1149_MOESM1_ESM.docx]

Dear Editor of *Journal of Ovarian Research*,

I will be much grateful if you consider and evaluate our manuscript titled *“Evaluation of the Relationship between Ovarian Reserve with Congenital Anomalies and Uterine Intermural Myomas: A Cross-Sectional Study”* for publication in your journal.

I confirm that this manuscript is original and has not been published elsewhere nor is currently under consideration for publication elsewhere.

The aim of this study is Evaluation of the Relationship between Ovarian Reserve markers with Congenital Anomalies and Uterine Intermural Myomas.

There are no conflicts of interests by any of the authors in this article. All authors are aware that they are being listed, have seen and approved the manuscript, and accept responsibility for its content.

Also the local Ethics Committee approval was obtained, as mentioned in the manuscript.

I look forward to hearing from you at your earliest convenience.

Sincerely,

Shahideh Jahanian Sadatmahalleh, Ph.D.

Assistant Professor

Department of Reproductive Health & Midwifery

Faculty of Medical Sciences

Tarbiat Modares University

Al-e-Ahmad Highway, Tehran, Iran, PO Box: 14115- 111

Tel.: (+98) 2182884826	; Fax: +98-21-82884560

E-mail: [Shahideh.Jahanian@modares.ac.ir](mailto:Shahideh.Jahanian@modares.ac.ir)

Maryam Niknejadi M.D.

Department of Reproductive Imaging, Reproductive Biomedicine Research Center

Royan Institute for Reproductive Biomedicine, ACECR

Royan institute

Resalat highway, end of North Bani Hashem St., East Hafez St.Tehran, Iran.

Al-e-Ahmad Highway, Tehran, Iran, PO Box: 14115- 111

Postal code: 1665659911	Tel: (+98) 2123562446	Fax: (+98) 2122306481

E-mail: [m.niknejadi@royaninstitute.org](mailto:m.niknejadi@royaninstitute.org)
